# Complete chloroplast genome of *Angiopteris yunnanensis* (Marattiaceae)

**DOI:** 10.1080/23802359.2019.1688111

**Published:** 2019-11-08

**Authors:** Qiuyu Jiang, Harald Schneider, Hongmei Liu

**Affiliations:** a Center for Integrative Conservation, Xishuangbanna Tropical Botanical Garden, Chinese Academy of Sciences, Menglun, China;; b University of Chinese Academy of Sciences, Beijing, China;; c Xishuangbanna Tropical Botanical Garden, Chinese Academy of Sciences, Menglun, China

**Keywords:** Christensenia, conservation genetics, evolutionary conservatism, marattioid ferns, molecular living fossil

## Abstract

The complete chloroplast genome of *Angiopteris yunnanensis* has been sequenced and assembled to provide genomic resources to reconstruct the phylogenetic relationship among species of *Angiopteris* as required to support conservation management of these ancient ferns. The chloroplast genome is 152,962 bp in length with the large single copy (LSC), the small single copy (SSC), and two inverted repeat (IR) regions of length 89,717bp, 20,585bp, and 21,330bp, respectively. We annotated 136 genes in total, including 89 coding genes, 39 tRNAs and 8 rRNAs. Comparative analyses confirmed the conservatism of plastid genome sequences among the species of *Angiopteris* and the distant related genus *Christensenia*.

*Angiopteris yunnanensis* Hieron. belongs to the pan-tropical family Marattiaceae, which is an ancient lineage without close extant relatives (Murdock 2008; PPG1 2016; Rothwell et al. 2018; Liu et al. 2019). The genus *Angiopteris* Hoffm. occurs mainly in tropical Asia and forms one of the most species rich genera of extant marattioid ferns (Murdock 2008; He and Christenhusz 2013). The taxonomic status of several local *Angiopteris* species is uncertain, which requires to be addressed using both nuclear and organelle genomes. Previously, the complete plastid sequences were published for two species of *Angiopteris* namely *Angiopteris evecta* (G.Forst.) Hoffm. (Roper et al. 2007) and *Angiopteris angustifolia* C.Presl (Zhu et al. 2016). Most recently the plastid genome of the marattioid genus *Christensenia* has been published (Liu et al. 2019). Previous phylogenetic studies were able to recover the generic relationships of *Angiopteris* but not the intrageneric relationships within this genus using a single coding nuclear gene (see Liu et al. 2019).

Here, we report the complete chloroplast genome sequence of *A. yunnanensis* (MN508633), a species restricted in its occurrence to Karst formations in Guangxi, Yunnan, and Northern Vietnam (He and Christenhusz 2013). The newly generated plastid genome was compared to the two previously published plastid genomes of *Angiopteris* and *Christensenia* using a statistical approach and phylogenetic analyses.

In this study, *A. yunnanensis* was sampled from Xishuangbanna Tropical Botanical Garden, Yunnan, China. A voucher specimen (Liu-CP05) was deposited in the Herbarium of Xishuangbanna Tropical Botanical Garden, Chinese Academy of Sciences (HITBC). The DNA sequence was made available via Genbank under the accession number MN508633. Total genomic DNA was extracted from 10–50 mg leaves using EasyPure Plant Genomic DNA kit (Transgen, Beijing, China). Genomic sequencing was generated on the Illumina HiSeq 2000 Platform (Illumina, Inc. San Diego, CA, USA). With the data obtained, raw reads were trimmed by Trimmomatic (Bolger et al. 2014), de novo assembly and confirmation were carried out by Getorganelle (Jin et al. 2018). The genome was annotated using PGA (Qu et al. 2019) and adjusted manually on Geneious 8.1.3 (Biomatters Ltd., Auckland, New Zealand).

The complete chloroplast genome of *A. yunnanensis* was 152,962 bp long with the typical quadripartite structure consisting of a pair of inverted repeat regions (IRs with 21,330 bp), divided by two single-copy regions (LSC with 89,717 bp; SSC with 20,585 bp). The overall GC content of the total length, LSC, SSC, and IR region was 35.40, 33.70, 32.90, and 40.40%, respectively. The genome contained 136 gene regions of which 86 coding genes, 32 tRNAs, and 4 rRNAs were unique. Comparison with the published genomes of marattioid ferns showed evidence for structural conservatism among the three *Angiopteris* and the single *Christensenia* genome, which is consistent with the hypotheses Marattiaceae as molecular living fossils (Soltis et al. 2002; Liu et al. 2019).

To determine the phylogenetic relationships of *A. yunnanensis*, the newly obtained sequence was integrated into a matrix including *A. angustifolia* [KP099647], *A. evecta* [DQ821119], and *Christensenia aesculifolia* (Blume) Maxon [MN056350] as in-group taxa, and the following outgroup taxa: *Equisetum hyemale* L. [KC117177], *Osmundastrum cinnamomeum* (L.) C.Presl [KF225592], *Ophioglossum californium* Prantl [KC117178]. *Psilotum nudum* (L.) P.Beauv. [AP004638]. 53 coding genes were randomly selected and assembled and aligned into a single sequence matrix. Model selection and maximum likelihood phylogeny tree were reconstructed using IQ-TREE (Nguyen et al. 2015).

Three species of *Angiopteris* formed a clade, whereas *Christensenia* was recovered as the sister to *Angiopteris.* The newly generated plastid sequence of *A. yunnanensis* suggests closer relationships to *A. evecta* than to *A. angustifolia* but the remarkable short branches indicated relatively little genetic differentiation among the three species of *Angiopteris*. This is consistent with the hypothesis of Marattiaceae as molecular fossils (Soltis et al. 2002; Liu et al. 2019). Together the four whole plastid genomes support the hypothesis of structural conservatism of chloroplast genomes in Marattiaceae (Roper et al. 2007; Zhu et al. 2016; Liu et al. 2019). The complete plastome sequence of *A. yunnanensis* will provide a useful resource for the conservation genetics of this species as well as for the phylogenetic studies for Marattiaceae (Figure 1).

**Figure 1.**
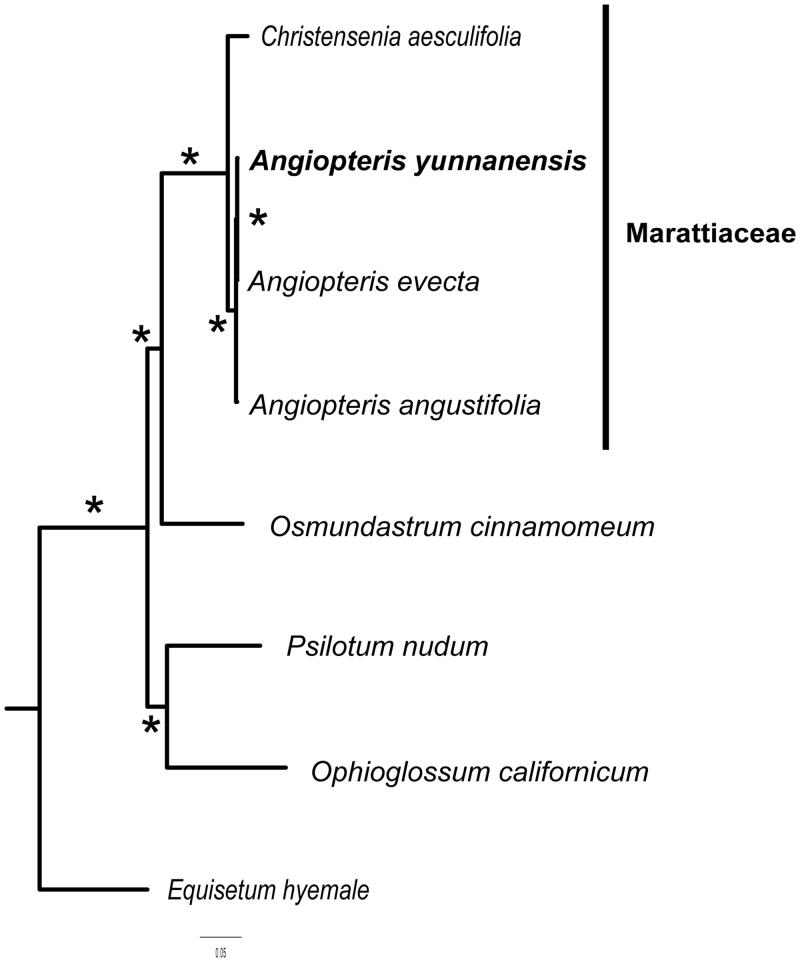
The best ML phylogeny tree recovered from 8 chloroplast genome by RAxML. Stars above branches correspond to bootstrap values of 100%. The sampling included four species belonging to the family Marattiaceae including besides the newly obtained plastid sequence of *Angiopteris yunnanensis* two other species of the genus *Angiopteris* and one species of *Christensenia*. As outgroup taxa, we included one representative of each of the four other major lineages of ferns.

## References

[CIT0001] Bolger AM, Lohse M, Usadel B 2014 Trimmomatic: a flexibel trimmer for Illumina sequence data. Bioinformatics. 30(15):2114–2120. 24695404 10.1093/bioinformatics/btu170 PMC4103590

[CIT0002] He ZR, Christenhusz M 2013 Marattiaceae Vols. 2–3 (Pteridophytes). In: Wu ZY, Raven PH, Hong DY, editors. Flora of China. Beijing: Science Press; p. 83–86.

[CIT0003] Jin JJ, Yu WB, Yang JB, Song Y, Yi TS, Li DZ 2018 GetOrganelle: a simple and fast pipeline for de novo assembly of a complete circular chloroplast genome using genome skimming data. bioRxiv. 2018:256479.

[CIT0004] Liu HM, Schneider H, Yu Y, Fujiwara T, Khine PK 2019 Towards the conservation of the Mesozoic relict fern *Christensenia*—a fern species with extremely small populations in China. J Plant Res. 132(5):601–616. 31446516 10.1007/s10265-019-01131-9

[CIT0005] Murdock AG 2008 Phylogeny of marattioid ferns (Marattiaceae): inferring a root in the absence of a closely related outgroup. Am J Bot. 95(5):626–641. 21632388 10.3732/ajb.2007308

[CIT0006] Nguyen LT, Schmidt HA, von Haeseler A, Minh BQ 2015 IQ-Tree: a feast and effective stochastic algorithm for estimating maximum-likelihood phylogenies. Mol Biol Evol. 32(1):268–274. 25371430 10.1093/molbev/msu300 PMC4271533

[CIT0007] PPG1. 2016 A community derived classification for extant lycophytes and ferns. J Syst Evol. 54:563–603.

[CIT0008] Qu XJ, Moore MJ, Li DZ, Yi TS 2019 PGA: a software package for rapid, accurate, and flexible batch annotation of plastomes. Plant Methods. 15:50. 31139240 10.1186/s13007-019-0435-7 PMC6528300

[CIT0009] Roper JM, Kellon Hansen S, Wolf PG, Karol KG, Mandoli DF, Everett KDE, Kuehl J, Boore JL 2007 The complete plastid genome sequence of *Angiopteris evecta* (G.Forst.) Hoffm. (Marattiaceae). Am Fern J. 97(2):95–106.

[CIT0010] Rothwell GW, Millay M, Stockey RA 2018 Resolving the overall pattern of marattialean fern phylogeny. Am J Bot. 105(8):1304–1314. 30001474 10.1002/ajb2.1115

[CIT0011] Soltis PS, Soltis DE, Savolainen V, Crane PR, Barraclough TG 2002 Rate heterogeneity among lineages of tracheophytes: integration of molecular and fossil data and evidence for molecular living fossils. Proc Natl Acad Sci USA. 99(7):4430–4435. 11917101 10.1073/pnas.032087199 PMC123665

[CIT0013] Zhu AD, Guo WH, Gupta S, Fan WH, Mower JP 2016 Evolutionary dynamics of the plastid inverted repeat: the effects of expansion, contraction, and loss on substitution rates. New Phytol. 209(4):1747–1175. 26574731 10.1111/nph.13743

